# Interplay between adipose tissue secreted proteins, eating behavior and obesity

**DOI:** 10.1007/s00394-021-02687-w

**Published:** 2021-10-12

**Authors:** Marleen Würfel, Jana Breitfeld, Claudia Gebhard, Markus Scholz, Ronny Baber, Steffi G. Riedel-Heller, Matthias Blüher, Michael Stumvoll, Peter Kovacs, Anke Tönjes

**Affiliations:** 1 Department of Medicine III, Division of Endocrinology, Nephrology and Rheumatology, University of Leipzig, Liebigstr. 18, 04103 Leipzig, Germany; 2 Helmholtz Institute for Metabolic, Obesity and Vascular Research (HI-MAG), Helmholtz Center Munich at the University of Leipzig and the University of Leipzig Medical Center, Leipzig, Germany; 3 Institute for Medical Informatics, Statistics and Epidemiology, Medical Faculty, University of Leipzig, Leipzig, Germany; 4 LIFE Research Centre for Civilization Diseases, Leipzig, Germany; 5 Medical Faculty, Institute of Social Medicine, Occupational Health and Public Health (ISAP), University of Leipzig, Leipzig, Germany; 6 German Center for Diabetes Research (DZD), Neuherberg, Germany

**Keywords:** Adipokines, Cytokines, Eating behavior, Obesity

## Abstract

**Purpose:**

Adipokines may play an important role in the complex etiology of human obesity and its metabolic complications. Here, we analyzed the relationship between 15 adipokines, eating behavior and body-mass index (BMI).

**Methods:**

The study included 557 participants of the Sorbs (62.1% women, 37.9% men) and 3101 participants of the population-based LIFE-Adult cohorts (53.4% women, 46.4% men) who completed the German version of the Three-Factor-Eating Questionnaire to assess the eating behavior types cognitive restraint, disinhibition and hunger. Serum levels of 15 adipokines, including adiponectin, adipocyte fatty acid-binding protein (AFABP), angiopoietin-related growth factor (AGF), chemerin, fibroblast growth factor (FGF)-19, FGF-21, FGF-23, insulin-like growth factor (IGF)-1, interleukin (IL) 10, irisin, progranulin, vaspin, pro-neurotensin (pro-NT), pro-enkephalin (PENK) and leptin were measured. Based on significant correlations between several adipokines with different eating behavior items and BMI, we conducted mediation analyses, considering the eating behavior items as potential mediation variable towards BMI.

**Results:**

Here, we found that the positive association between chemerin, AFABP or leptin and BMI in Sorbian women was mediated by higher restraint or disinhibited eating, respectively. Additionally, in Sorbian women, the negative relation between IGF-1 and BMI was mediated by higher disinhibition and the positive link between AGF and BMI by lower disinhibition. In Sorbian men, the negative relationship between PENK and BMI was mediated by lower disinhibition and hunger, whereas the negative relation between IGF-1 and BMI was mediated by higher hunger. In the LIFE-Adult women´s cohort, associations between chemerin and BMI were mediated by decreased hunger or disinhibition, respectively, whereas relations between PENK and BMI were fully mediated by decreased disinhibition.

**Conclusion:**

Our study suggests that adipokines such as PENK, IGF-1, chemerin, AGF, AFABP and leptin might affect the development of obesity by directly modifying individual eating behavior. Given the observational nature of the study, future experimental or mechanistic work is warranted.

**Supplementary Information:**

The online version contains supplementary material available at 10.1007/s00394-021-02687-w.

## Introduction

The global prevalence of overweight and obesity is continuously increasing and has become one of the major global health burdens [1]. Compared to people with normal weight, patients with overweight and obesity suffer more frequently from metabolic diseases like type 2 diabetes, dyslipidemia and hypertension, resulting in an elevated risk for coronary heart disease, pulmonary diseases, stroke, obstructive sleep apnea and different types of cancer [2]. The development of obesity is a complex process mediated by lifestyle factors like food intake and a lack of physical activity, as well as a genetic predisposition. Furthermore, some pharmacotherapies, endocrine and mental disorders contribute to an increased risk of obesity [3].

It has been recognized that adipose tissue is not only an energy storage, but also an endocrine organ producing hundreds of secretory proteins, so-called adipokines, which are involved in a variety of biological processes [4]. Adipokines are bioactive peptides secreted by adipose tissue and interacting with various organs, e.g. the brain, vasculature, muscle, pancreas and liver. Central actions of adipokines include the regulation of appetite and eating behavior, insulin secretion, inflammation, lipid metabolism, blood pressure or reproduction [5].

Alterations in adipose tissue belong to the primary defects in obesity. Caused by genetic, environmental and behavioral factors such as individual eating behavior, adipose tissue dysfunction may link adiposity to metabolic, inflammatory and cardiovascular diseases. It is known that increased energy intake leads to hyperplasia and hypertrophy of adipocytes causing cellular stress and inflammatory response in adipose tissue and other organs. It is also well acknowledged, that the inflammatory properties of adipokines play an important role in inducing obesity-related disorders [6]. Therefore, it is important to understand the complex relationships between modifiable factors like eating behavior and adipokines in the pathophysiology of human obesity.

Recent findings demonstrate associations between adipokines and various states of obesity [5, 7, 8]. Exemplarily, several studies suggested vaspin’s protective role against insulin resistance in state of obesity [9–11]. Moreover, intracerebroventricular vaspin administration resulted in reduced food intake and blood glucose in mice [12]. Findings of another study confirmed the hypothesis that vaspin plays a role in the cognitive control of food intake by pointing out associations between vaspin serum levels and different types of eating behavior [11]. Briefly, serum vaspin concentrations positively correlated with restraint, disinhibition and hunger after adjustments for age and BMI. In addition, a potential association between progranulin serum levels and eating behavior was proposed [13]. Accordingly, higher progranulin serum levels in patients with obesity obviously result in progranulin resistance. Consequently, the abolished anorexigenic effect of progranulin further leads to increased appetite and food intake [14]. However, these studies comprised several limitations within the single study designs like small sample sizes and the inclusion of subjects with comorbidities like diabetes mellitus which themselves could possibly bias the outcome. To overcome these limitations and to improve the understanding of the relationships between adipokines and eating behavior in the pathophysiology of obesity, the present study investigated the associations between 15 adipokines supposed to be involved in peripheral as well as central regulatory mechanisms (adiponectin, adipocyte fatty acid-binding protein (AFABP), angiopoietin-related growth factor (AGF), chemerin, fibroblast growth factor (FGF)-19, FGF-21, FGF-23, insulin-like growth factor (IGF)-1, interleukin (IL) 10, irisin, progranulin, vaspin, pro-neurotensin (pro-NT), pro-enkephalin (PENK) and leptin) and eating behavior assessed by the German version of the self-rating Three-Factor-Eating-Questionnaire (TFEQ) in two metabolically well-characterized cohorts: the LIFE-Adult (*N* = 3,101) [15] and the Sorbs cohort (*N* = 557) [16].

## Materials and methods

### Subjects

#### Sorbs cohort

Subjects included in this study have been extensively metabolically phenotyped between 2005 and 2007 as described elsewhere [16]. The TFEQ was available for 557 participants without diabetes aged 18–82 years. The recruitment of subjects was carried out population based without special inclusion criteria aside from Sorbian origin based on self-reported family history. Exclusion criteria were age below 18 years, pregnancy or lactation period and acute infections. For the present analyses, we furthermore excluded subjects suffering from diabetes from the dataset.

#### LIFE-Adult cohort

LIFE-Adult is a population-based study of 10,000 inhabitants of the city of Leipzig, Germany. All participants underwent a broad range of clinical examinations, interviews and standardized questionnaires which were conducted by trained study personal at the LIFE research center in the University Hospital of Leipzig (see [15] for details).

Out of the total cohort, 3101 subjects between 19 and 80 years with completed phenotyping and according to our exclusion criteria (diabetes mellitus, thyroid diseases, malignant diseases, and medication interfering with glucose homeostasis) were selected for further analysis.

### German version of the Three-Factor-Eating Questionnaire (TFEQ)

Eating behavior was assessed by the German version of the TFEQ “Fragebogen zum Essverhalten” (FEV) including 51 items to derive three dimensions of eating behavior, namely: cognitive restraint, disinhibition and hunger [17]. The item “restraint” originally consists of 21 questions and describes the ability of permanent cognitive control to regulate food intake and consequently body weight. In contrast, the domain “disinhibition” includes 16 items that define the loss of permanent cognitive control over food intake because of various factors such as emotional states or different external circumstances. Additional 14 items represent “hunger” as the exaggerated feeling of hunger or the urge to take food.

### Protein measurements

Serum concentrations of all adipokines under investigation (adiponectin, chemerin, FGF-19/21/23, progranulin, AFABP, vaspin, IL-6, leptin, PENK, irisin, AGF) were determined with a commercially available enzyme-linked immunosorbent assay (ELISA) according to the manufacturer’s instructions (AGF, irisin, vaspin and progranulin: AdipoGen Inc, Seoul, Korea; FGF-19, FGF-21, adiponectin, chemerin and AFABP: BioVendor Inc, Brno, Czech Republic; IL-6: R&D Systems, Minneapolis, MN USA; leptin: Mediagnost, Reutlingen, Germany; FGF-23: Immutopics, San Clemente, CA, USA). PENK and pro-NT was centrally quantified in a single lab by sphingotec (sphingotec GmbH, Hennigsdorf, Germany) using a chemiluminometric sandwich immunoassay [18]. Serum IGF-1 concentrations were measured by commercially available automated two-site chemiluminescent immunometric assays (Immulite 2000, Siemens Healthcare Diagnostics GmbH, Bad Nauheim, Germany). Whereas all adipokines were measured in the Sorbs cohort, only a subset of them (chemerin, vaspin, pro-NT, PENK) was recorded in the LIFE-Adult cohort. Therefore, the more comprehensively phenotyped Sorbs cohort was taken for the primary analyses, whereas the replication analyses of four adipokines were performed in the larger LIFE-Adult cohort.

### Statistical analyses

Statistical analyses were performed using the statistical software package IBM SPSS Statistics Version 24.0 for Windows, including the use of the SPSS macro PROCESS v3.3 by Andrew F. Hayes [19] for mediation analysis. For our analyses, a P value < 0.05 was considered to be statistically significant. Bonferroni correction was used to account for multiple comparisons, but due to the absence of eating behavior phenotypes in the majority of reported studies, we decided to provide also borderline significant associations/correlations to allow the readers to make their own interpretation about the strength of the reported correlations/associations.

We performed descriptive analyses for anthropometric and metabolic measurements presented as median [interquartile range] and stratified by gender. Differences were tested using the Mann–Whitney *U* test. Furthermore, the three types of eating behavior cognitive restraint, disinhibition and hunger were compared between men and women by Chi-square test. To analyze different levels of the eating behavior, we used five categories from “very low” to “very high” [17]. Spearman´s rank correlation was used to analyze the correlation between adipokine serum levels and eating behavior, including additionally age and BMI.

Furthermore, linear regression analysis was used to identify the relationship of age, eating behavior and adipokine serum levels on BMI values. In our gender-stratified regression model, we included the BMI as the dependent and sum scores of the TFEQ factors (cognitive restraint, disinhibition, hunger), the 15 adipokine serum levels and age as independent variables. Normal distribution of the residuals was checked and confirmed visually by inspection of the P–P plots.

Based on results of our previous correlation and linear regression analyses, we conducted gender-stratified mediation analysis to consider possible interactions between various types of eating behavior, BMI and adipokine serum levels. For our mediation analysis, we postulated that variations in eating behavior (cognitive restraint, disinhibition and hunger) mediate the relation between different serum levels of adipokines and BMI. We analyzed a simple mediation model to examine direct, indirect and total effects of eating behavior on the correlation between adipokines and BMI. For this mediation model, one adipokine was thought to present an impact on eating behavior leading thereby to an impact on BMI. Thus, the adipokine was the independent variable, the eating behavior (disinhibition or hunger or cognitive restraint) was the mediator and the BMI, the dependent variable. In our mediation analysis, a direct effect presents a direct relation between one adipokine and BMI. An indirect effect comprises the product of the path between the adipokine and BMI, on one side, and between one adipokine, mediator and BMI, on the other side. The total effect describes the sum of direct and indirect effect. Consequently, a full mediation effect was assumed if there was an indirect effect via altered eating behavior but no direct effect of one adipokine on BMI. Otherwise, if the path between adipokine and BMI was as significant as the path between adipokine, eating behavior and BMI, there was only a partial mediation effect. To uncover relations between variables, mediation analysis with bootstrapping procedure of 5000 samples providing 95% CI was used. The SPSS macro by Preacher and Hayes was used with Model 4, including the Sobel Test for testing the significance of the direct and indirect effects.

## Results

### Baseline characteristics

#### Sorbs cohort

Among the 557 studied Sorbs (62.1% women and 37.9% men), women had a median age of 45 [34–54] years and a median BMI of 24.8 [22.0–28.4] kg/m^2^. Men had a median age of 46 [32–55] years and a median BMI of 26.2 [24.4–28.1] kg/m^2^.

We observed significant differences between women and men for BMI, waist-to-hip ratio (WHR), fat mass (%), lean body mass (LBM), fasting plasma glucose (FG), high-density lipoprotein cholesterol (HDL-C), low-density lipoprotein cholesterol (LDL-C), triglycerides (TG) and the serum levels of adiponectin, AFABP, chemerin, FGF-19, FGF-23, irisin, vaspin, PENK and leptin (all *P* ≤ 0.01) (Table 1). Whereas the eating behavior score hunger did not significantly differ between the both groups (*P* = 0.237), there were gender-specific differences in the cognitive restraint (*P* < 0.001) and disinhibition (*P* < 0.001) domains. In comparison to men, women were more often represented in the “higher” and “very high” scores of the item disinhibition (11.9% vs 3.8%). Moreover, women also reached higher scores in the category cognitive restraint in contrast to men (45.8% vs 21.8%) (Table 2).

**Table 1:** Baseline characteristics of the Sorbs population (without diabetic subjects; *N* = 557)

	Women	Men	*P* value
*Anthropometric parameters*			
Age (years)	45.35 [33.85–53.80]	45.70 [31.85–54.68]	0.382
BMI (kg/m^2^)	24.80 [22.00–28.38]	26.20 [24.43–28.08]	**0.001**
WHR	0.80 [0.75–0.86]	0.94 [0.86–0.99]	** < 0.001**
*Adipokines*			
Adiponectin (mg/l)	18.41 [14.75–21.40]	13.40 [10.14–18.08]	** < 0.001**
AFABP (µg/l)	18.63 [11.33–29.50]	11.82 [6.97–16.73]	** < 0.001**
AGF (µg/l)	38.93 [24.41–57.84]	43.10 [25.21–59.74]	0.296
Chemerin (µg/l)	119.79 [95.54–142.30]	103.53 [86.23–126.05]	**0.001**
FGF-19 (ng/l)	195.6 [131.6–330.2]	246.6 [160.7–387.5]	**0.033**
FGF-21 (ng/l)	57.98 [15.26–126.72]	76.58 [29.52–153.21]	0.096
FGF-23 (RU/ml)	74.30 [59.83–104.64]	68.80 [57.17–87.32]	** < 0.001**
IGF-1 (µg/l)	163.40 [113.85–203.20]	170.85 [128.48–203.00]	0.618
IL-10 (ng/l)	4.16 [1.88–10.31]	5.03 [2.10–9.31]	0.628
Irisin (mg/l)	0.83 [0.66–1.04]	0.74 [0.61–0.94]	**0.017**
Progranulin (µg/l)	105.90 [89.14–124.94]	103.70 [90.17–124.14]	0.919
Vaspin (µg/l)	0.67 [0.30–1.60]	0.34 [0.18–0.53]	** < 0.001**
pro-NT (pmol/l)	113.66 [88.39–148.61]	108.60 [76.60–144.74]	0.681
PENK (pmol/l)	59.01 [49.14–68.80]	55.10 [46.67–63.75]	** < 0.001**
Leptin (ng/µl)	15.57 [9.01–26.08]	4.82 [3.22–7.87]	** < 0.001**

Values were stratified by gender and provided as median [interquartile range]. Variables were analyzed by Mann–Whitney *U* test

*AFABP* adipocyte fatty acid-binding protein, *AGF* angiopoietin-related growth factor, *BMI* body-mass index, *FG* fasting glucose, *FGF* fibroblast growth factor, *FI* fasting insulin, *HbA1c* glycated hemoglobin A1c, *HDL-C* high-density lipoprotein cholesterol, *HOMA-IR* homeostasis model assessment of insulin resistance, *IGF-1* insulin-like growth factor, *IL* interleukin, *LBM* lean body mass, *LDL-C* low-density lipoprotein cholesterol, *N* number, *TG* triglycerides, *WHR* waist-to-hip ratio, *pro-NT* pro-neurotensin, *PENK* pro-enkephalin

**Table 2:** Eating behavior phenotypes of the Sorbs cohort

Scoring within phenotype	Disinhibition		Hunger		Cognitive restraint	
	Women (*N*)	Men (*N*)	Women (*N*)	Men (*N*)	Women (*N*)	Men (*N*)
Very low	139	106	118	92	68	58
Low	82	64	94	54	40	64
Median	76	31	60	34	72	36
High	23	4	46	22	80	30
Very high	17	4	19	7	72	14
*P* value	**0.001**		0.237		** < 0.001**	

Variables were analyzed by χ^2^ test and stratified by gender (*N*_women_ = 346/ *N*_men_ = 211). *N* numbers

#### LIFE-Adult cohort

Among the 3101 participants of the LIFE-Adult cohort (53.4% women and 46.4% men), women had a median BMI of 26.4 [23.7–29.8] kg/m^2^, whereas men had a median BMI of 27.0 [24.9–29.6] kg/m^2^. The median age of the studied women was 64 [55–71] years and of the men 65 [55–72] years.

We identified significant differences between genders for BMI, WHR as well as for serum levels of the adipokines: chemerin, vaspin and PENK (all *P* < 0.001) (Table 3).

**Table 3:** Baseline characteristics of the LIFE-Adult cohort (*N* = 3101)

	Women	Men	*P* value
*Anthropometric parameters*			
Age (years)	64 [55–71]	65 [55–72]	**0.011**
BMI (kg/m^2^)	26.40 [23.71–29.80]	26.97 [24.91–29.62]	** < 0.001**
WHR	0.88 [0.84–0.92]	1.00 [0.96–1.04]	** < 0.001**
*Adipokines*			
Chemerin (µg/l)	168 [149–191]	157 [139–178]	** < 0.001**
Vaspin (µg/l)	0.32 [0.19–0.61]	0.21 [0.12–0.39]	** < 0.001**
pro-NT (pmol/l)	107.51 [83.99–138.00]	106.49 [80.7–138.09]	0.258
PENK (pmol/l)	61.99 [52.86–73.04]	57.61 [48.43–67.89]	** < 0.001**

Values were stratified by gender and provided as median [interquartile range]. Variables were analyzed by Mann–Whitney *U* test

*BMI* body-mass index, *WHR* waist-to-hip ratio, *pro-NT* pro-neurotensin, *PENK* pro-enkephalin

Likewise, we noticed significant gender-dependent differences in all three eating behavior items (all *P* < 0.001) (Table 4). Compared with men, the proportion of women was significantly higher in categories “high” and “very high” (disinhibition: 7.3% *vs.* 2.4%, hunger: 4.6% *vs.* 1.9%, cognitive restraint: 45.5% *vs.* 26.9%).

**Table 4:** Eating behavior phenotypes of the LIFE-Adult cohort

Scoring within phenotype	Disinhibition		Hunger		Cognitive restraint	
	Women (*N*)	Men (*N*)	Women (*N*)	Men (*N*)	Women (*N*)	Men (*N*)
Very low	420	558	572	664	125	232
Low	298	291	219	202	168	235
Median	132	61	93	61	184	190
High	59	21	41	18	216	157
Very high	8	1	2	0	182	85
*P *value	** < 0.001**		** < 0.001**		** < 0.001**	

Variables were analyzed by χ^2^ test and stratified by gender (*N*_women_ = 1657/ *N*_men_ = 1444)

### Correlation between adipokine serum levels, eating behavior and BMI

In the first step, we conducted Spearman´s rank correlation analysis to understand and identify connections between adipokines and eating behavior. In the second step, we examined the relationship of both, adipokine levels and eating behavior with BMI.

#### Sorbs cohort

*Adipokines and eating behavior:* Correlations between adipokine serum levels and the eating behavior domain disinhibition were found for serum levels of IGF-1 correlating positively in men and women (Table 5), whereas a correlation in only one gender was found for AGF (*ρ* = -0.157) and leptin (*ρ* = 0.160) in women and PENK (*ρ* = -0.230) in men (Table 5).

**Table 5:** Spearman´s rank correlation in the Sorbs cohort (*N* = 557)

	Score disinhibition				Score hunger				Score cognitive restraint				BMI			
	Women		Men		Women		men		Women		Men		Women		Men	
	*P* value	*ρ *	*P* value	*ρ *	*P* value	*ρ *	*P* value	*ρ *	*P* value	*ρ *	*P* value	*ρ *	*P* value	*ρ *	*P* value	*ρ *
***Anthropometric parameters***																
Age	**6.68 x 10**^-7^*	– 0.324	**0.001**	– 0.229	**1.20 x 10**^-7^*	– 0.280	**0.002**	– 0.214	**0.007**	0.145	**0.052**	0.134	**5.76 x 10**^-21^*	0.478	**5.00 x 10**^-10^*	0.414
BMI	**0.031**	0.117	**0.003**	0.203	**0.031**	– 0.116	**0.029**	0.152	**7.00 x 10**^-6^*	0.239	0.106	0.112				
***Adipokines***																
Adiponectin	0.114	– 0.089	0.222	– 0.087	0.466	– 0.041	**0.034**	– 0.151	0.744	0.018	0.055	0.137	**0.003**	– 0.169	0.293	-0.076
AFABP4	0.634	– 0.026	0.557	– 0.042	0.084	– 0.093	0.747	– 0.023	**4.97 x 10**^-4^*	0.187	0.506	0.047	**1.00 x 10**^-39^*	0.635	**8.80 x 10**^-12^*	0.459
AGF	**0.008**	– 0.157	0.340	– 0.073	0.126	– 0.092	0.433	– 0.060	0.677	0.025	0.822	0.017	0.943	0.004	0.801	-0.020
Chemerin	0.767	0.016	0.413	– 0.058	0.638	– 0.026	**0.010**	– 0.180	**0.046**	0.108	0.506	0.047	**1.04 x 10**^-8^*	0.305	**0.005**	0.199
FGF-19	0.285	– 0.059	0.098	– 0.116	0.130	0.083	**0.031**	– 0.151	0.674	– 0.023	0.771	0.021	0.055	– 0.105	0.311	0.072
FGF-21	0.064	– 0.104	0.108	– 0.118	0.513	– 0.037	0.331	– 0.072	**0.032**	0.121	0.220	0.090	**4.36 x 10**^-8^*	0.303	**2.77 x 10**^-5^*	0.305
FGF-23	0.634	– 0.026	0.560	– 0.040	0.558	– 0.032	0.537	– 0.043	0.190	0.071	0.405	0.058	0.929	0.005	0.779	– 0.020
IGF-1	**4.33 x 10**^-4^*	0.188	**0.042**	0.140	**9.40 x 10**^-5^*	0.209	**0.001**	0.233	0.432	– 0.042	0.448	– 0.052	**1.46 x 10**^-14^*	– 0.400	**7.79 x 10**^-5^*	– 0.270
IL-10	0.970	– 0.002	0.260	0.079	0.105	0.090	0.763	0.021	0.599	0.029	0.813	– 0.017	0.160	– 0.079	0.572	0.040
Irisin	0.412	0.044	0.807	0.017	0.985	– 0.001	0.106	– 0.112	0.529	0.034	0.188	0.091	0.535	– 0.034	**0.006**	– 0. 190
Progranulin	0.085	– 0.093	0.486	0.048	0.964	– 0.002	0.463	– 0.051	0.440	– 0.042	**0.048**	-0.136	0.073	0.097	0.905	0.008
Vaspin	0.129	0.082	0.610	– 0.035	0.264	0.060	0.828	– 0.015	0.792	0.014	0.557	0.041	**0.007**	– 0.145	**0.007**	0.187
Pro-NT	0.167	– 0.075	0.245	– 0.081	0.697	– 0.021	**0.004**	– 0.196	0.506	– 0.036	0.780	-0.019	0.883	– 0.008	0.057	– 0.133
PENK	0.099	– 0.089	**0.001**	– 0.230	0.760	0.017	**0.006**	– 0.190	0.119	– 0.084	0.811	0.017	**1.05 x 10**^-10^*	– 0.341	**1.09 x 10**^-6^*	– 0.331
Leptin	**0.007**	0.160	0.697	0.028	0.657	– 0.027	0.598	– 0.038	**4.30 x 10**^-4^*	0.209	0.996	0.000	**1.04 x 10**^-43^*	0.710	**1.81 x 10**^-13^*	0.498

Data were stratified by gender. Data present P values and Spearman´s correlation coefficients (*ρ)*. *P* values < 0.05 were considered to be significant and, therefore, marked in bold. *P* values which were significant after Bonferroni correction were additionally marked with *

*AFABP* adipocyte fatty acid-binding protein, *AGF* angiopoietin-related growth factor, *BMI* body mass index, *FGF* fibroblast growth factor, *IGF-1* insulin-like growth factor, *IL*, interleukin, *N* number, *pro-NT* pro-neurotensin, *PENK*, pro-enkephalin

Regarding correlations of the item hunger and adipokines, we were able to identify a positive correlation of IGF-1 in both genders (Table 5), whereas adiponectin, chemerin, FGF-19, pro-NT and PENK correlated negative in men only (Table 5). The third eating item “cognitive restraint” does not present any correlation the same in both genders. Here, we detected a negative correlation with progranulin in men (Table 5) and positive correlations with AFABP4, chemerin, FGF-21 and leptin in women (Table 5).

*Adipokines and BMI:* We found statistically significant positive correlations of serum levels of AFABP4, chemerin, FGF-21 and leptin with BMI for men and women. Additionally, IGF-1 and PENK serum levels correlated negatively with BMI. When examining differences between women and men, we observed a significant negative correlation with adiponectin serum levels and BMI for the entire cohort (data not shown) which was mainly driven by the correlation in women (Table 5), whereas the correlation between irisin and BMI in men (Table 5) did not appear in the whole cohort (data not shown). Moreover, we noticed a positive correlation between serum levels of vaspin and BMI in men, whereas it was negative in women (Table 5).

*BMI and eating behavior:* Finally, we found various correlations between BMI and different types of eating behavior: in men and women. Thus, BMI was positively correlated with the item disinhibition in both genders, whereas cognitive restraint correlated positively in women only. This correlation was also not seen in the entire Sorbs cohort (data not shown). Moreover, BMI correlated positively with the score of hunger in men, but negatively in women (Table 5) and was, therefore, abolished in the whole cohort (data not shown).

#### LIFE-Adult cohort

*Adipokines and eating behavior:* Regarding the correlation between adipokines and the eating behavior item disinhibition we detected a significant negative correlation in both genders for PENK only (Table 6). Additionally, we found a positive correlation of vaspin in men and of chemerin in women (Table 6). For the item hunger, the only significant correlation was found with chemerin (ρ = 0.079) in women (Table 6) and the eating type “cognitive restraint” was positively correlated with vaspin in men only (Table 6).

**Table 6:** Spearman´s rank correlation in the LIFE-Adult cohort (*N* = 3101)

	Score disinhibition				Score hunger				Score cognitive restraint				BMI			
	Women		Men		Women		Men		Women		Men		Women		Men	
	*P* value	*ρ*	*P* value	*ρ*	*P* value	*ρ*	*P* value	*ρ*	*P* value	*ρ*	*P* value	*ρ*	*P* value	*ρ*	*P* value	*ρ*
*Anthropometric parameters*																
Age	**1.66 × 10**^–6^*	– 0.157	**1.06 × 10**^–11^*	– 0.220	0.013*	– 0.081	**4.41 × 10**^–4^*	– 0.114	**1.92 × 10**^–8^*	0.189	**2.30 × 10**^–19^*	0.294	**3.50 × 10**^–9^*	0.145	0.198	0.034
BMI	**7.00 × 10**^–30^*	0.363	**4.61 × 10**^–19^*	0.294	**2.89 × 10**^–8^*	0.181	**3.40 × 10**^–5^*	0.134	**5.07 × 10**^–9^*	0.196	0.007*	0.090				
*Adipokines*																
Vaspin	0.729	– 0.012	0.009*	0.087	0.249	0.039	0.144	0.048	0.684	0.014	0.007*	0.091	0.831	–0.005	**3.40 × 10**^–9^*	0.157
Chemerin	**3.62 × 10**^–4^*	0.118	0.290	– 0.035	0.017*	0.079	0.699	0.013	0.191	0.044	0.801	– 0.008	**5.24 × 10**^–71^*	0.418	**1.33 × 10**^–26^*	0.281
pro-NT	0.909	– 0.004	0.799	– 0.008	0.071	– 0.060	0.193	0.042	0.697	– 0.013	0.639	– 0.016	**0.032**	0.053	0.174	0.036
PENK	**1.90 × 10**^–4^*	– 0.123	**5.20 × 10**^–5^*	– 0.132	0.256	– 0.038	0.647	– 0.015	0.080	0.059	0.157	0.047	**0.007**	–0.066	**3.40 × 10**^–10^*	–0.165

Data were stratified by gender. Data present *P* values and Spearman´s correlation coefficients (*ρ)*. *P* values < 0.05 were considered to be significant and therefore marked in bold. *P* values which were significant after Bonferroni correction were additionally marked with *

*BMI* body-mass index, *N* number, *pro-NT* pro-neurotensin, *PENK* pro-enkephalin

*Adipokines and BMI:* We observed a statistically significant positive correlation between chemerin and BMI and a negative correlation between PENK and BMI in both genders (Table 6). Furthermore, while pro-NT was positively associated with BMI in women only, vaspin showed a positive correlation with BMI in men (Table 6). Both correlations have not been seen in the entire LIFE-Adult cohort (data not shown).

*BMI and eating behavior:* When examining the correlation between eating behavior and BMI, all three items correlated with BMI, in both women and men (Table 6).

### Linear regression analysis

To refine the relationship between individual BMI with different adipokine serum levels and eating behavior, we applied age adjusted, gender-stratified linear regression analyses for each single cohort. In addition, we compared the analyzed effects between the both cohorts.

#### Sorbs cohort

In both genders, we found higher BMI values significantly associated with higher scores of disinhibition, higher serum levels of AFABP4 and leptin, as well as lower serum levels of PENK (Table 7). Furthermore, age, FGF-21 and IL-10 were predictors of higher BMI, in men only. Additionally, higher values of BMI were related to lower levels of adiponectin and pro-NT in male subjects, whereas in women, higher BMI was significantly associated with lower levels in IGF-1 and vaspin serum concentrations (Table 7).

**Table 7:** Gender-stratified linear regression analysis in the Sorbs cohort

	BMI					
	Men (*N* = 211)			Women (*N* = 346)		
	β	Standard error	*P* value	β	Standard error	*P* value
*Anthropometric parameter*						
Age	0.068	0.016	** < 0.001**	0.041	0.022	0.059
*Eating behavior*						
Disinhibition	0.337	0.097	**0.001**	0.328	0.086	** < 0.001**
Hunger	– 0.047	0.092	0.614	– 0.108	0.094	0.250
Cognitive restraint	0.099	0.050	0.051	– 0.005	0.045	0.915
*Adipokines*						
Adiponectin	– 0.121	0.037	**0.001**	– 0.001	0.044	0.990
AFABP4	0.059	0.029	**0.042**	0.087	0.015	** < 0.001**
AGF	0.008	0.006	0.185	– 0.003	0.007	0.687
Chemerin	0.008	0.007	0.239	– 0.003	0.005	0.514
FGF-19	7.892 × 10^–5^	0.001	0.922	0.001	0.001	0.517
FGF-21	0.002	0.000	**0.001**	0.000	0.001	0.857
FGF-23	– 0.002	0.006	0.731	0.000	0.001	0.813
IGF-1	0.004	0.004	0.290	– 0.009	0.005	**0.043**
IL-10	0.021	0.009	**0.019**	– 0.002	0.006	0.714
Irisin	– 1.096	0.725	0.134	0.267	0.766	0.728
Progranulin	– 0.009	0.008	0.279	0.000	0.007	0.948
Vaspin	– 0.110	0.203	0.590	– 0.272	0.130	**0.038**
pro-NT	– 0.013	0.004	**0.004**	0.001	0.004	0.889
PENK	– 0.055	0.017	**0.001**	– 0.051	0.011	** < 0.001**
Leptin	0.135	0.046	**0.004**	0.164	0.017	** < 0.001**

*P* values were calculated by linear regression analysis. The analysis was conducted for BMI as dependent variable and age, eating behavior and adipokine serum levels as independent variables. *P* values < 0.05 were considered to be significant and therefore marked in bold. *AFABP* adipocyte fatty acid-binding protein, *AGF* angiopoietin-related growth factor, *BMI* body-mass index, *FGF* fibroblast growth factor, *IGF-1* insulin-like growth factor, *IL* interleukin, *pro-NT* pro-neurotensin, *PENK* pro-enkephalin, *N* number, *β*  regression coefficient

#### LIFE-Adult cohort

Within the LIFE-Adult cohort, linear regression analysis revealed that in both genders, higher individual BMI values were significantly associated with disinhibited eating behavior, higher age, as well as higher chemerin and lower PENK levels (Table 8). In this analysis, only women presented a positive relation between higher BMI values and scores of cognitive restrained eating (Table 8).

**Table 8:** Gender-stratified linear regression analysis in the LIFE-Adult cohort

	BMI					
	Men (*N* = 1444)			Women (*N* = 1657)		
	β	Standard error	*P* value	β	Standard error	*P* value
*Anthropometric parameter*						
Age	0.044	0.011	** < 0.001**	0.070	0.014	** < 0.001**
*Eating behavior*						
Disinhibition	0.548	0.062	** < 0.001**	0.525	0.062	** < 0.001**
Hunger	– 0.047	0.060	0.432	– 0.065	0.068	0.334
Cognitive restraint	0.030	0.027	0.258	0.097	0.030	**0.001**
*Adipokines*						
Chemerin	0.028	0.004	** < 0.001**	0.049	0.004	** < 0.001**
Vaspin	0.355	0.181	0.050	– 0.267	0.165	0.106
pro-NT	0.001	0.002	0.713	– 0.004	0.003	0.137
PENK	– 0.014	0.004	** < 0.001**	– 0.031	0.008	** < 0.001**

*P* values were calculated by linear regression analysis. The analysis was conducted for BMI as dependent variable and age, eating behavior and adipokine serum levels as independent variables. *P* values < 0.05 were considered to be significant and therefore marked in bold. *BMI* body-mass index, *pro-NT* pro-neurotensin, *PENK* pro-enkephalin, *N* number, *β *regression coefficient

#### Sorbs- vs. LIFE-Adult cohort

Comparing the eight overlapping traits (age, three eating behavior items, four adipokines) which were available in both cohorts and which were analyzed within this study, we found the same effects in both cohorts for increased BMI and higher age in male subjects and increased disinhibition scores, and lower PENK levels in both genders (Tables 7 and 8). The remaining traits presented differences comparing both cohorts with significant association in one of the two cohorts, only: for chemerin in both genders and age, and cognitive restraint in females of the LIFE-Adult cohort; and pro-NT in male Sorbs. The effect directions for all overlapping significant associations were the same in both cohorts (Tables 7 and 8).

### Mediation analysis

To clarify whether eating behavior was the mediator of the relationship between adipokine serum levels and BMI as the dependent outcome of the model, mediation analyses were conducted. The mediation models were based on the results of correlation and linear regression analyses as described above, with only significant relationships being considered for mediation.

#### Sorbs cohort

Since several adipokines were significantly correlated to various types of eating behavior (without Bonferroni correction) in our correlation analysis (Table 5), we performed gender-stratified mediation analyses for each of these adipokines. We found a significant direct effect between PENK and BMI in both genders (both: *P* < 0.001; Online Resource 1), whereas the indirect effect via the mediator disinhibition was significant in men only (*P* < 0.01; Online Resource 1b); thus, suggesting a partial mediation in men but not in women (Table 9; Online Resource and Online Resource 1a, 1b). The same result was seen for the mediator hunger. Here, also in both genders, a significant direct effect was found between the adipokine PENK and BMI (both: *P* < 0.001 Online Resource 2), whereas the indirect effect via the mediator hunger was significant in men only (*P* < 0.05; Online Resource 2b). Thus, also the item hunger, at least partially, impacts the BMI in men, but not in women (Table 9; Online Resource 2 and Online Resource 2a, 2b).

**Table 9:** Overview of significant mediation models within the Sorbs and LIFE-Adult cohorts

Mediation	Eating behavior phenotype	Sorbs cohort (Women/Men)	LIFE-Adult (Women/Men)
Partial	Cognitive restraint	chemerin + /– AFABP + /–	/
	Disinhibition	AGF + /– IGF-1 + /– PENK –/ + leptin + /–	chemerin + /–
	Hunger	PENK –/ + IGF-1 –/ +	chemerin + /–
Full	Disinhibition	/	PENK + /–

The mediation model was defined as followed: independent variable = adipokine; mediator = eating behavior; dependent variable = BMI

*AFABP* adipocyte fatty acid- binding protein, *AGF* angiopoietin-related growth factor, *IGF-1* insulin-like growth factor, *PENK* pro-enkephalin, *BMI* body-mass index

+ , significant; –, not significant

Regarding the other adipokines, we found partial mediation for IGF-1 and the item hunger in men only (*P* < 0.001; Table 9; Online Resource 3; Online Resource 3b). In women, we found a partial mediation for IGF-1, AGF and leptin via the item disinhibition (Table 9; Online Resource 4, 5 and 6; Online Resource 4a, 5a, 6a), and chemerin and AFABP via the item cognitive restraint (all *P* < 0.05; Table 9; Online Resource 7 and 8, Online Resource 7a and 8a).

#### LIFE-Adult cohort

Following the results of the correlation and linear regression analyses, we constructed mediation models for the adipokines chemerin, PENK and vaspin (see above; Tables 6 and 8).

Consequently, we developed a mediation model for chemerin as the independent, the item hunger as the mediator, and BMI as the dependent variable. In both men and women, there was a significant direct path between chemerin and BMI. However, whereas the indirect path between chemerin and BMI via the mediator hunger was statistically significant in women (*P* < 0.01), but not in men (Table 9; Online Resource 9, Online Resource 9a, 9b).

Furthermore, we found comparable results conducting the mediation chemerin—eating item disinhibition—BMI. Also, within this model, we detected a partial mediation in women but not in men (Table 9, Online Resource 10 and Online Resource 10a).

Regarding the other correlations, the model including PENK as independent, the item disinhibition as mediator and BMI as dependent variable was the only one presenting a full mediation by the absence of a direct effect (*P* = 0.34) between PENK and BMI but a significant indirect effect via PENK—disinhibition—BMI (*P* < 0.001) in women (Table 9; Online Resource 11, Online Resource 11a). This model appears to be the most robust, since all statistical models presented a relationship of disinhibition—PENK—BMI.

Result of mediation models with P > 0.05 are presented in the Supplementary material 1 only (Online Resource 12–22 and Online Resource 12-22a, 12-22b).

## Discussion

The aim of this study was to investigate associations between serum levels of adipokines and eating behavior items to suggest their potential influence on food intake. Therefore, we used the TFEQ to assess the eating behavior in two independent cohorts, the Sorbs and the LIFE-Adult cohort.

In the present study, higher scores of disinhibition were associated with higher BMI values in female and male participants of the LIFE-Adult and Sorbs cohorts. Whereas we identified inconclusive results in regard to the manifestation of restraint eating between both genders. Thus, higher scoring in cognitive restraint was a predictor for higher BMI in women of the LIFE-Adult cohort, but also provided a tendency to be increased in male Sorbs. Whereas it is an established assumption, that restraint eating is often positive associated with increased body weight and higher BMI [20, 21], as we could also see in our data, the interpretation of the cause or consequence of restrained eating still remains unclear. It is reported, that restrained eaters are more vulnerable for binge eating tendencies, resulting in higher BMI values [22]. However, the positive association between this eating behavior trait and BMI might be explained by the motivation to use restraint eating tendencies to prevent weight gain in obese individuals [23]. Restrained eating is often differently expressed between men and women, whereas women were supposed to use restrained eating strategies more frequently than men with the motivation to reduce food intake and weight gain [24].

To analyze whether adipokines have impact on the eating behavior phenotype in human cohorts, we investigated correlations between their serum levels and BMI as well as eating behavior in a first step, followed by the analysis of various mediation models to test the potential role of eating behavior in the link between adipokines and BMI.

In the past, the adipokine chemerin has attracted attention as a potential link between obesity and its comorbidities, most likely mediated by chemerin’s role in inflammatory processes [25, 26]. Beyond the previously reported positive correlation with BMI [25, 27–29], our analyses hint to a role of chemerin in the regulation of eating behavior. Based on the observed correlations between chemerin and the eating behavior phenotypes, cognitive restraint, hunger and disinhibition mediation analyses suggested that chemerin could play a role in the cognitive control of eating behavior and food intake which results in an increased BMI. These findings are in line with recent studies describing the role of chemerin in a neuroendocrine axis [30–32]. Among others, they delineate the existence of chemerin and its receptor CMKLR1 in different brain regions, such as hypothalamus, hippocampus or prefrontal cortex [30]. Furthermore, acute intracerebroventricular administration of chemerin into rats may lower BMI temporary, whereas chronic chemerin infusion increased food intake and body weight [30]. Finally, gender dimorphism of chemerin should be noted, as majority, but not all reported studies indicate higher chemerin levels in women [25, 26, 33, 34]. It is, therefore, possible that the lack of causal relationships between chemerin, eating behavior and BMI in men reflects the strong gender specificity of this adipokine, thus deserving further extensive research.

In line with other studies, our analyses revealed correlations between serum levels of the white adipose tissue derived”satiety hormone” leptin and higher BMI values [35–37]. Leptin physiologically acts, among other important brain regions, in the hypothalamic Nucleus arcuatus via the inhibition of orexigenic Neuropeptide Y- and Agouti-Related Protein-expressing neurons and activates the anorectic POMC neurons [31]. Whereas under normal conditions leptin mediates satiety and facilitates energy expenditure, enlarged adipocytes in obese individuals were supposed to cause hyperleptinemia, which results in leptin resistance, along going with a desensitization of leptin-mediated impacts [38, 39]. Consistent with previous observations that leptin may further increase BMI via affecting uncontrolled eating patterns, under certain metabolic conditions, our study revealed positive correlations between leptin concentrations and scores of disinhibition and cognitive restraint in women. Significant results of mediation analyses between leptin, the eating behavior item disinhibition and BMI supported the thesis that long-term leptin overexpression attenuate leptins anorexigenic effects, which may again result in increased food intake and appetite, as well as lower energy expenditure and body weight gain. Thus, Benbaibeche et al. outlined leptin as a biomarker for uncontrolled eating in the way that the combination of dysfunctional eating behavior and hyperleptinemia leads to an resistance of leptin-mediated satiety [40]. Furthermore, the gender dimorphism which we detected by significant associations between leptin concentrations and eating behavior subtypes was also evident in leptin serum values. Accordingly, previous studies also showed higher circulating leptin concentrations in women compared to men [36, 41, 42]. Whereas exact causative mechanisms for this gender discrepancy still remains unclear, Mantzoros and Moschos summarized several reasons like the influence of sex steroids, the higher female sensitivity to other hormones like insulin or glucocorticoids and the widely discussed influence of the differences in female and male fat distribution [43].

Furthermore, in the present study, lower PENK levels were associated with increased BMI, which was in line with negative correlations between PENK and scores of disinhibition and hunger. Subsequent mediation analyses suggested that PENK might support overeating which results in elevated BMI. Interestingly, PENK is supposed to belong to the members of endogenous opioids acting in the reward system. Kelley et al. revealed that especially endogenous opioids in the Nucleus accumbens play a key role in mediating the palatability of consumed food [44]. In support of our findings, downregulation of *pro-enkephalin* gene expression occurred in rats with free access to palatable food [44, 45], which the authors explained by compensatory mechanisms emerging from long-term effects of fat- and sugar-induced higher food palatability [44]. Several studies suggested, that the treatment with opioid antagonists such as naloxone or naltrexone leads to reduced food consumption through a decreased palatability (reviewed in [46]). In addition, increased fat intake was observed in rats injected with an μ-opioid-receptor agonist [D-Ala^2^, N-;Me-Phe^4^, Gly^5^-ol]-Enkephalin (DAMGO) [47, 48]. Furthermore, results indicate that the consumption of palatable food increased the acute expression of central acting endogenous opioids, which again resulted in higher food intake [46]. In rats, the gene expression and synthesis of *enkephalin* and *dynorphin* increased in the hypothalamic Nucleus paraventricularis with the consumption of fat, but at this time independent of a higher food palatability [49], possibly caused by direct effects of circulating triglycerides on the neuronal opioid expression. In line with this finding, Kim et al. showed a reduction of food amount decreased the gene expression of *pro-dynorphine*, *pro-enkephalin* and *proopiomelanocortin* in hypothalamic Nucleus arcuatus [50]. Altogether, despite a solid evidence for the opioid modulation of palatable food intake, only little is known about the alterations in central acting opioids in relation to eating behaviour. Specific mechanisms linking the endogenous opioid synthesis with food consumption thus still remain unclear.

The adipokine AFABP was positively correlated with BMI in our present study, which was consistent with previously published data showing a relationship between higher levels of serum AFABP and obesity-related metabolic irregularities [51–55]. Our findings including a positive correlation between AFABP levels and the eating behavior subtype cognitive restraint imply that AFABP may influence our eating behavior in various ways, resulting in uncontrolled food intake and in consequence in weight gain and higher BMI. Although actions of this adipokine in relation to eating behavior are scarcely studied, previous research showed that body weight loss intervention like physical exercise and bariatric surgery could significantly reduce circulating AFABP levels [55]. Moreover, chemical inhibitors of AFABP render beneficial effects against disorders associated with features of the metabolic syndrome like insulin resistance or atherosclerosis [56]. Whether eating behavior may act as a potential mediator between AFABP and metabolic diseases as suggested by the present mediation analyses, remains to be determined in larger prospective epidemiological studies.

Out of the investigated adipokines in our study, we would like to discuss two more proteins in detail. Thus, we found no gender differences in serum levels of AGF and a negative correlation with disinhibition. This is supported by George who presented AGF as a member of the angiopoietin-like protein (Angptl) family and described that increased serum levels of AGF (Angptl6) lead to significant weight loss and an improved insulin sensitivity [57]. Given the observed partial mediation for the pathways between AGF, disinhibition and BMI, we propose that higher AGF concentrations may diminish overeating which could ultimately result in changes in BMI. Our assumption is in line with Oike et al. who showed that AGF treatment significantly lowered body weight in mice suggesting its role in states of obesity and insulin resistance by regulating energy metabolism [58]. Nevertheless, they did not detect any differences in daily food intake between the AGF-treated and control group.

As a part of the Growth Hormone (GH)/IGF-1/Insulin axis, we also investigated IGF-1 and found a significant negative correlation between serum levels of IGF-1 and BMI. Whereas some researchers reported elevated IGF-1 levels in obese compared with lean or overweight people [59], several studies presented lower IGF-1 serum levels in association with higher fat mass [60, 61] and components of the metabolic syndrome [62]. As proposed by Savastano et al., this inconsistency could be explained by the feedback system of the GH/IGF-1 axis and its secretory mechanisms which is affected by several maladaptive mechanisms including elevations of circulating free fatty acids, adipokines or cytokines due to increased visceral adipose tissue or other ectopic fat depots finally leading to diminished IGF-1 bioactivity (reviewed in [60]). Supporting this hypothesis, the authors provided evidence, that recombinant human growth hormone (rhGH) treatment in obese women had favorable effects after bariatric surgery as it reduced body weight through a loss of fat mass, improved lipid profile and insulin sensitivity [63]. In the context of eating behavior, we detected positive correlations of IGF-1 with disinhibition and hunger. This is in contrast to Fujita et al. who showed that intracerebroventricular administration of IGF-1 significantly decreased food intake in chicken [64]. However, Hawkes et al. highlighted that the IGF-1 concentration is sensitive to alterations in nutritional status and pointed out that over-nutrition tends to increase GH- and IGF-1 sensitivity which leads to obesity-related diseases [65]. These data including ours, provoke further research under consideration of various mediation models to better understand the mechanistic chains between eating behavior traits, IGF-1 and BMI.

In summary, this is the first study analyzing the associations of 15 adipokines with eating behavior and obesity. Despite the relatively large sample size for studying associations including adipokines and eating behavior, we still see limitations in the statistical power for testing causal relationships. Although two independent cohorts were included in the study, validation in larger cohorts providing better statistical power is warranted. It cannot be ruled out that adipokines mediate the effects of eating behavior on BMI. This would, however, require evidence for the role of eating behavior in regulating adipokine levels, which is currently missing. It is of note as well, that the physiological rationale for some of the lesser studied adipokines is scarce to support the here studied direction and, therefore, reverse causality, i.e. changes in the production of these adipokines may be secondary to obesity, could also be true.

Furthermore, in our study, we aimed to identify relationships between biological quantities and eating behavior. Eating behavior is a complex concept which is operationalized in our study by scoring of a questionnaire, i.e. there is no direct measurable biological quantity related to this trait. Due to this simplification of a complex neurological concept, strong correlations cannot be expected to our opinion. This would require identifying neurophysiological equivalents of eating behavior.

Moreover, analyzed traits are complex, i.e. are affected by a multitude of parameters. Therefore, it is unlikely that single predictors explain large proportions of the phenotype variances. In this regard, we consider correlations > 0.1 (or < -0.1) as relevant. Sample size of our study implies that such correlations achieve nominal significance in our analyses.

## Conclusion

Concerning the scarcely studied central influence of adipokines on human eating behavior and metabolic diseases, our study may open new paths and initiate further work on the role of adipokines in the complex etiology of human adiposity. Our study suggests that adipokines such as pro-enkephalin, IGF-1, chemerin, AGF, AFABP and leptin may affect obesity by directly controlling eating behavior (expressed as disinhibition, cognitive restraint and hunger). Without doubt, future extensive research is warranted to specify the diverse mechanisms of adipokines in weight gain potentially via the neuroendocrine axis, which may ultimately contribute to the development of adipokine-related innovative approaches in treatment of obesity.

## Supplementary Information

Below is the link to the electronic supplementary material.

Supplementary file1 (DOCX 422 KB)

## Data Availability

All data are available in the manuscript and the according supplemental material or will be provided upon request.
