# Supplementary material for: Interplay between adipose tissue secreted proteins, eating behavior and obesity
Source: Eur J Nutr. 2021 Oct 12;61(2):885–99. doi: 10.1007/s00394-021-02687-w (PMC8854280; doi:10.1007/s00394-021-02687-w)
Supplement: Supplementary file 1 — Supplementary file1 (DOCX 422 KB) [file 394_2021_2687_MOESM1_ESM.docx]

**Online Resource**

**Interplay between adipose tissue secreted proteins, eating behavior and obesity**

**Journal:** European Journal of Nutrition

**Authors:** Würfel M^*^, Breitfeld J^*^, Gebhard C, Scholz M, Baber R, Riedel-Heller SG, Blüher M, Stumvoll M, Kovacs P, Tönjes A^#^

^#^ Corresponding author:

Anke Tönjes, MD,

Department of Medicine III,

Division of Endocrinology, Nephrology and Rheumatology,

University of Leipzig,

Liebigstr. 18, 04103 Leipzig, Germany.

Phone: +49 341 97-13380,

Email: [Anke.toenjes@medizin.uni-leipzig.de](mailto:Anke.toenjes@medizin.uni-leipzig.de)

The mediation model was defined as followed:

independent variable= adipokine;

mediator= eating behavior type;

dependent variable= BMI

Numbers of subjects within the analyses for the single cohorts:

| cohort | all | women | men |
| --- | --- | --- | --- |
| Sorbs | 557 | 346 | 211 |
| LIFE Adult | 3101 | 1657 | 1444 |

The Structure of the following pages is the same for all:

X table providing the effect size, standard error, P-value, and confidence interval

Xa figure of the mediation model for the females

Xb figure of the mediation model for the males

1. PENK – disinhibition – Sorbs cohort
2. PENK – hunger – Sorbs cohort
3. IGF-1 – hunger – Sorbs cohort
4. IGF-1 – disinhibition – Sorbs cohort
5. AGF – disinhibition – Sorbs cohort
6. leptin – disinhibition – Sorbs cohort
7. chemerin – cognitive restraint – Sorbs cohort
8. AFABP – cognitive restraint – Sorbs cohort
9. chemerin – hunger – LIFE Adult cohort
10. chemerin – disinhibition – LIFE Adult cohort
11. PENK – disinhibition – LIFE Adult cohort
12. chemerin – hunger – Sorbs cohort
13. chemerin – disinhibition – Sorbs cohort
14. FGF-19 – hunger – Sorbs cohort
15. pro-NT – hunger – Sorbs cohort
16. adiponectin – hunger – Sorbs cohort
17. FGF-21 – cognitive restraint – Sorbs cohort
18. progranulin – cognitive restraint – Sorbs cohort
19. leptin – cognitive restraint – Sorbs cohort
20. PENK – hunger – LIFE Adult cohort
21. vaspin – disinhibition – LIFE Adult cohort
22. vaspin – cognitive restraint – LIFE Adult cohort

**Online Resource 1**: Mediation model for pro-enkephalin (PENK) and “disinhibition” on BMI for women and men of the Sorbs cohort.

|  | **b** | **SE** | **P value** | **Bootstrap 95% CI** | |
| --- | --- | --- | --- | --- | --- |
| ***Women*** |  |  |  |  |  |
| Total effect | -0.0874 | 0.0137 | <0.001 | -0.1142 | -0.0605 |
| Direct effect | -0.0839 | 0.0135 | <0.001 | -0.1106 | -0.0573 |
| Indirect effect | -0.0034 | 0.0032 |  | -0.0114 | 0.0012 |
| ***Men*** |  |  |  |  |  |
| Total effect | -0.0909 | 0.0174 | <0.001 | -0.1251 | -0.0566 |
| Direct effect | -0.0788 | 0.0176 | <0.001 | -0.1136 | 0.0441 |
| Indirect effect | -0.0121 | 0.0061 |  | -0.0257 | -0.0019 |

b=effect size; SE=standard error; CI=confidence interval

PENK

BMI

Score_disinhibition

a=- 0.0150

b= 0.2283 **

c= -0.0874***

c´= -0.0839*

**Online Resource 1a**: Mediation model between PENK, eating behavior trait “disinhibition“ and BMI for **women** of the Sorbs cohort.

* P< 0.05, ** P< 0.01, *** P< 0.001; c= total effect; c´= direct effect; a, b= regression coefficient.

PENK

BMI

Score_disinhibition

a= -0.0485 ***

b= 0. 2487**

c= -0.0909 ***

c´= -0.0788 ***

**Online Resource 1b**: Mediation model between PENK, eating behavior trait “disinhibition“ and BMI for **men** of the Sorbs cohort.

* P< 0.05, ** P< 0.01, *** P< 0.001; c= total effect; c´= direct effect; a, b= regression coefficient.

**Online Resource 2**: Mediation model between PENK and “hunger” on BMI for women and men of the Sorbs cohort.

|  | **b** | **SE** | **P value** | **Bootstrap 95% CI** | |
| --- | --- | --- | --- | --- | --- |
| ***Women*** |  |  |  |  |  |
| Total effect | -0.0874 | 0.0137 | <0.001 | -0.1142 | -0.0605 |
| Direct effect | -0.0872 | 0.0137 | <0.001 | -0.1142 | -0.0603 |
| Indirect effect | -0.0001 | 0.0011 |  | -0.0027 | 0.0023 |
| ***Men*** |  |  |  |  |  |
| Total effect | -0.0909 | 0.0174 | <0.001 | -0.1251 | -0.0566 |
| Direct effect | -0.0838 | 0.0176 | <0.001 | -0.1185 | -0.0491 |
| Indirect effect | -0.0071 | 0.0054 |  | -0.0200 | 0.0009 |

b=effect size; SE=standard error; CI=confidence interval

PENK

BMI

Score_hunger

0.0103

-0.0114

c= -0.0874 ***

c´= -0.0872***

**Online Resource 2a**: Mediation model between PENK, eating behavior trait “hunger“ and BMI for **women** of the Sorbs cohort.

* P< 0.05, ** P< 0.01, *** P< 0.001; c= total effect; c´= direct effect; a, b= regression coefficient.

PENK

BMI

Score_hunger

-0.0403 **

0.1752 *

c= -0.0909 ***

c´= -0.0838***

**Online Resource 2b**: Mediation model between PENK, eating behavior trait “hunger“ and BMI for **men** of the Sorbs cohort.

* P< 0.05, ** P< 0.01, *** P< 0.001; c= total effect; c´= direct effect; a, b= regression coefficient.

**Online Resource 3**: Mediation model between insulin-like growth factor (IGF)-1 and “hunger” on BMI for women and men of the Sorbs cohort.

b=effect size; SE=standard error; CI=confidence interval

|  | **b** | **SE** | **P value** | **Bootstrap 95% CI** | |
| --- | --- | --- | --- | --- | --- |
| ***Women*** |  |  |  |  |  |
| Total effect | -0.0277 | 0.0039 | <0.001 | -0.0354 | 0.0200 |
| Direct effect | -0.0284 | 0.0040 | <0.001 | -0.0362 | -0.0205 |
| Indirect effect | 0.0007 | 0.0009 |  | -0.0008 | 0.0027 |
| ***Men*** |  |  |  |  |  |
| Total effect | -0.0160 | 0.0038 | <0.001 | -0.0234 | -0.0085 |
| Direct effect | -0.0197 | 0.0037 | <0.001 | -0.0271 | -0.0123 |
| Indirect effect | 0.0037 | 0.0015 |  | 0.0012 | 0.0070 |

IGF-1

BMI

Score_hunger

a= 0. 0088***

b= 0.0759

c= -0.0277***

c´= -0.0284***

**Online Resource 3a**: Mediation model between IGF-1, eating behavior trait “hunger“ and BMI for **women** of the Sorbs cohort.

* P< 0.05, ** P< 0.01, *** P< 0.001; c= total effect; c´= direct effect; a, b= regression coefficient.

IGF-1

BMI

Score_hunger

a= 0.0103 ***

b= 0. 3621***

c= -0.0160 ***

c´= -0.0197 ***

**Online Resource 3b**: Mediation model between IGF-1, eating behavior trait “hunger“ and BMI for **men** of the Sorbs cohort.

* P< 0.05, ** P< 0.01, *** P< 0.001; c= total effect; c´= direct effect; a, b= regression coefficient.

**Online Resource 4**: Mediation model between IGF-1 and “disinhibition” on BMI for women and men of the Sorbs cohort.

|  | **b** | **SE** | **P value** | **Bootstrap 95% CI** | |
| --- | --- | --- | --- | --- | --- |
| ***Women*** |  |  |  |  |  |
| Total effect | -0.0277 | 0. 0039 | <0.001 | -0.0354 | -0.0200 |
| Direct effect | -0.0309 | 0.0038 | <0.001 | -0.0385 | -0.0234 |
| Indirect effect | 0.0032 | 0.0013 |  | 0.0011 | 0.0061 |
| ***Men*** |  |  |  |  |  |
| Total effect | -0.0160 | 0.0038 | <0.001 | -0.0234 | -0.0085 |
| Direct effect | -0.0182 | 0.0036 | <0.001 | -0.0254 | -0.0110 |
| Indirect effect | 0.0022 | 0.0013 |  | 0.0001 | 0.0053 |

b=effect size; SE=standard error; CI=confidence interval

IGF-1

BMI

Score_disinhibition

a= 0.0083 **

b= 0.3887***

c= -0.0277***

c´=-0.0309***

**Online Resource 4a**: Mediation model between IGF-1, eating behavior trait “disinhibition“ and BMI for **women** of the Sorbs cohort.

* P< 0.05, ** P< 0.01, *** P< 0.001; c= total effect; c´= direct effect; a, b= regression coefficient.

IGF-1

BMI

Score_disinhibition

a= 0.0056

b= 0.4001***

c= -0.0160***

c´= -0.0182 ***

**Online Resource 4b**: Mediation model between IGF-1, eating behavior trait “disinhibition“ and BMI for **men** of the Sorbs cohort.

* P< 0.05, ** P< 0.01, *** P< 0.001; c= total effect; c´= direct effect; a, b= regression coefficient.

**Online Resource 5**: Mediation model between angiopoietin-related growth factor (AGF) and “disinhibition” on BMI for women and men of the Sorbs cohort.

|  | **b** | **SE** | **P value** | **Bootstrap 95% CI** | |
| --- | --- | --- | --- | --- | --- |
| ***Women*** |  |  |  |  |  |
| Total effect | 0.0178 | 0.0101 | 0.0782 | -0.0020 | 0.0377 |
| Direct effect | 0.0232 | 0.0100 | 0.0215 | 0.0034 | 0.0429 |
| Indirect effect | -0.0053 | 0.0027 |  | -0.0111 | -0.0009 |
| ***Men*** |  |  |  |  |  |
| Total effect | 0.0058 | 0.0084 | 0.4920 | -0.0108 | 0.0224 |
| Direct effect | 0.0081 | 0.0082 | 0.3207 | -0.0080 | 0.0243 |
| Indirect effect | -0.0024 | 0.0023 |  | -0.0076 | 0.0018 |

b=effect size; SE=standard error; CI=confidence interval

AGF

BMI

Score_disinhibition

a= -0.0162**

b= 0.3295 ***

c= 0.0178

c´= 0.0232 **

**Online Resource 5a**: Mediation model between AGF, eating behavior trait “disinhibition“ and BMI for **women** of the Sorbs cohort.

* P< 0.05, ** P< 0.01, *** P< 0.001; c= total effect; c´= direct effect; a, b= regression coefficient.

AGF

BMI

Score_disinhibition

a= -0.0071

b= 0.3328 ***

c= 0.0058

c´= 0.0081

**Online Resource 5b**: Mediation model between AGF, eating behavior trait “disinhibition“ and BMI for **men** of the Sorbs cohort.

* P< 0.05, ** P< 0.01, *** P< 0.001; c= total effect; c´= direct effect; a, b= regression coefficient.

**Online Resource 6**: Mediation model between leptin and “disinhibition” on BMI for women and men of the Sorbs cohort.

b=effect size; SE=standard error; CI=confidence interval

|  | **b** | **SE** | **P value** | **Bootstrap 95% CI** | |
| --- | --- | --- | --- | --- | --- |
| ***Women*** |  |  |  |  |  |
| Total effect | 0.2444 | 0.0151 | <0.001 | 0.2147 | 0.2742 |
| Direct effect | 0.2394 | 0.0152 | <0.001 | 0.2096 | 0.2692 |
| Indirect effect | 0.0050 | 0.0040 |  | -0.0003 | 0.0150 |
| ***Men*** |  |  |  |  |  |
| Total effect | 0.1830 | 0.0362 | <0.001 | 0.1092 | 0.2569 |
| Direct effect | 0.1895 | 0.0396 | <0.001 | 0.1084 | 0.2706 |
| Indirect effect | -0.0064 | 0.0251 |  | -0.0747 | 0.0280 |

leptin

BMI

Score_disinhibition

a= 0.0337*

b= 0.1491*

c= 0.2444***

c´= 0.2394***

**Online Resource 6a:** Mediation model between leptin, eating behavior trait “disinhibition“ and BMI for **women** of the Sorbs cohort.

* P< 0.05, ** P< 0.01, *** P< 0.001; c= total effect; c´= direct effect; a, b= regression coefficient.

leptin

BMI

Score_disinhibition

a= 0.0533*

b= -0.1210

c= 0.1830***

c´= 0.1895***

**Online Resource 6b:** Mediation model between leptin, eating behavior trait “disinhibition“ and BMI for **men** of the Sorbs cohort.

* P< 0.05, ** P< 0.01, *** P< 0.001; c= total effect; c´= direct effect; a, b= regression coefficient.

**Online Resource 7**: Mediation model between chemerin and “cognitive restraint” on BMI for women and men of the Sorbs cohort.

|  | **b** | **SE** | **P value** | **Bootstrap 95% CI** | |
| --- | --- | --- | --- | --- | --- |
| ***Women*** |  |  |  |  |  |
| Total effect | 0.0326 | 0.0065 | <0.001 | 0.0197 | 0.0454 |
| Direct effect | 0.0301 | 0.0065 | <0.001 | 0.0174 | 0.0429 |
| Indirect effect | 0.0024 | 0.0014 |  | 0.0002 | 0.0056 |
| ***Men*** |  |  |  |  |  |
| Total effect | 0.0214 | 0.0068 | 0.0018 | 0.0081 | 0.0348 |
| Direct effect | 0.0211 | 0.0068 | 0.0021 | 0.0077 | 0.0345 |
| Indirect effect | 0.0003 | 0.0007 |  | -0.0011 | 0.0019 |

b=effect size; SE=standard error; CI=confidence interval

chemerin

BMI

Score_cognitive restraint

a= 0. 0149*

b= 0. 1642**

c= 0.0326***

c´= 0.0301***

**Online Resource 7a**: Mediation model between chemerin, eating behavior trait “cognitive restraint“ and BMI for **women** of the Sorbs cohort.

* P< 0.05, ** P< 0.01, *** P< 0.001; c= total effect; c´= direct effect; a, b= regression coefficient.

chemerin

BMI

Score_cognitive restraint

a= 0.0074

b= 0.0438

c= 0.0214 **

c´= 0.0211 **

**Online Resource 7b**: Mediation model between chemerin, eating behavior trait “cognitive restraint“ and BMI for men of the Sorbs cohort.

* P< 0.05, ** P< 0.01, *** P< 0.001; c= total effect; c´= direct effect; a, b= regression coefficient.

**Online Resource 8**: Mediation model between adipocyte fatty acid-binding protein (AFABP) and “cognitive restraint” on BMI for women and men of the Sorbs cohort.

b=effect size; SE=standard error; CI=confidence interval

|  | **b** | **SE** | **P value** | **Bootstrap 95% CI** | |
| --- | --- | --- | --- | --- | --- |
| ***Women*** |  |  |  |  |  |
| Total effect | 0.1788 | 0.0363 | <0.001 | 0.1074 | 0.2501 |
| Direct effect | 0.1739 | 0.0360 | <0.001 | 0.1031 | 0.2447 |
| Indirect effect | 0.0048 | 0.0027 |  | 0.0008 | 0.0109 |
| ***Men*** |  |  |  |  |  |
| Total effect | 0.1750 | 0.0229 | <0.001 | 0.1299 | 0.2200 |
| Direct effect | 0.1734 | 0.0237 | <0.001 | 0.1267 | 0.2201 |
| Indirect effect | 0.0016 | 0.0029 |  | -0.0039 | 0.0084 |

AFABP

BMI

Score_cognitive restraint

a= 0.0474*

b= 0.1018 *

c= 0.1788***

c´= 0.1739***

**Online Resource 8a:** Mediation model between AFABP, eating behavior trait “cognitive restraint“ and BMI for **women** of the Sorbs cohort.

* P< 0.05, ** P< 0.01, *** P< 0.001; c= total effect; c´= direct effect; a, b= regression coefficient.

AFABP

BMI

Score_cognitive restraint

a= 0. 0405

b= 0.0388

c= 0. 1750***

c´= 0. 1734***

**Online Resource 8b:** Mediation model between AFABP, eating behavior trait “cognitive restraint“ and BMI for **men** of the Sorbs cohort.

* P< 0.05, ** P< 0.01, *** P< 0.001; c= total effect; c´= direct effect; a, b= regression coefficient.

**Online Resource 9**: Mediation model between chemerin and “hunger” on BMI for women and men of the LIFE Adult cohort.

|  | **b** | **SE** | **P value** | **Bootstrap 95% CI** | |
| --- | --- | --- | --- | --- | --- |
| ***Women*** |  |  |  |  |  |
| Total effect | 0.0535 | 0.0062 | <0.001 | 0.0413 | 0.0657 |
| Direct effect | 0.0510 | 0.0060 | <0.001 | 0.0392 | 0.0628 |
| Indirect effect | 0.0025 | 0.0009 |  | 0.0009 | 0.0045 |
| ***Men*** |  |  |  |  |  |
| Total effect | 0.0266 | 0.0041 | <0.001 | 0.0185 | 0.0346 |
| Direct effect | 0.0264 | 0.0041 | <0.001 | 0.0184 | 0.0344 |
| Indirect effect | 0.0002 | 0.0005 |  | -0.0009 | 0.0012 |

b=effect size; SE=standard error; CI=confidence interval

chemerin

BMI

Score_hunger

a= 0.0081**

b= 0.3071***

c= 0.0535***

c´= 0.0510***

**Online Resource 9a:** Mediation model between chemerin, eating behavior trait “hunger“ and BMI for **women** of the LIFE Adult cohort.

* P< 0.05, ** P< 0.01, *** P< 0.001; c= total effect; c´= direct effect; a, b= regression coefficient.

chemerin

BMI

Score_hunger

a= 0.0007

b= 0.2169 ***

c= 0.0266 ***

c´=0.0264 ***

**Online Resource 9b:** Mediation model between chemerin, eating behavior trait “hunger“ and BMI for **men** of the LIFE Adult cohort.

* P< 0.05, ** P< 0.01, *** P< 0.001; c= total effect; c´= direct effect; a, b= regression coefficient.

**Online Resource 10**: Mediation model between chemerin and “disinhibition” on BMI for women and men of the LIFE Adult cohort.

|  | **b** | **SE** | **P value** | **Bootstrap 95% CI** | |
| --- | --- | --- | --- | --- | --- |
| ***Women*** |  |  |  |  |  |
| Total effect | 0.0540 | 0.0062 | <0.001 | 0.0418 | 0.0663 |
| Direct effect | 0.0484 | 0.0059 | <0.001 | 0.0369 | 0.0599 |
| Indirect effect | 0.0056 | 0.0017 |  | 0.0025 | 0.0091 |
| ***Men*** |  |  |  |  |  |
| Total effect | 0.0247 | 0.0041 | <0.001 | 0.0166 | 0.0328 |
| Direct effect | 0.0251 | 0.0039 | <0.001 | 0.0175 | 0.0327 |
| Indirect effect | -0.0004 | 0.0012 |  | -0.0028 | 0.0018 |

b=effect size; SE=standard error; CI=confidence interval

chemerin

BMI

Score_disinhibition

a= 0.0108***

b= 0.5224***

c= 0.0540***

c´= 0.0484***

**Online Resource 10a:** Mediation model between chemerin, eating behavior trait “disinhibition“ and BMI for **women** of the LIFE Adult cohort.

* P< 0.05, ** P< 0.01, *** P< 0.001; c= total effect; c´= direct effect; a, b= regression coefficient.

chemerin

BMI

Score_disinhibition

a= -0.0009

b= 0. 4564***

c=0.0247 ***

c´=0.0251 ***

**Online Resource 10b:** Mediation model between chemerin, eating behavior trait “disinhibition“ and BMI for **men** of the LIFE Adult cohort.

* P< 0.05, ** P< 0.01, *** P< 0.001; c= total effect; c´= direct effect; a, b= regression coefficient.

**Online Resource 11**: Mediation model between pro-enkephalin (PENK) and “disinhibition” on BMI for women and men of the LIFE Adult cohort.

|  | **b** | **SE** | **P value** | **Bootstrap 95% CI** | |
| --- | --- | --- | --- | --- | --- |
| ***Women*** |  |  |  |  |  |
| Total effect | -0.0204 | 0.0102 | 0.0447 | -0.0404 | -0.0005 |
| Direct effect | -0.0094 | 0.0098 | 0.3395 | -0.0286 | 0.0099 |
| Indirect effect | -0.0111 | 0.0028 |  | -0.0172 | -0.0058 |
| ***Men*** |  |  |  |  |  |
| Total effect | -0.0066 | 0.0087 | 0.4525 | -0.0237 | 0.0106 |
| Direct effect | -0.0048 | 0.0076 | 0.5243 | -0.0198 | 0.0101 |
| Indirect effect | -0.0017 | 0.0021 |  | -0.0084 | 0.0004 |

b=effect size; SE=standard error; CI=confidence interval

PENK

BMI

Score_disinhibition

a= -0.0190***

b= 0.5823***

c= -0.0204*

c´= -0.0094

**Online Resource 11a:** Mediation model between PENK, eating behavior trait “disinhibition“ and BMI for **women** of the LIFE Adult cohort.

* P< 0.05, ** P< 0.01, *** P< 0.001; c= total effect; c´= direct effect; a, b= regression coefficient.

PENK

BMI

Score_disinhibition

a= -0.0039

b= 0.4474***

c=-0.0066

c´=-0.0048

**Online Resource 11b:** Mediation model between PENK, eating behavior trait “disinhibition“ and BMI for **men** of the LIFE Adult cohort.

* P< 0.05, ** P< 0.01, *** P< 0.001; c= total effect; c´= direct effect; a, b= regression coefficient.

**Online Resource 12**: Mediation model between chemerin and “hunger” on BMI for women and men of the Sorbs cohort.

|  | **b** | **SE** | **P value** | **Bootstrap 95% CI** | |
| --- | --- | --- | --- | --- | --- |
| ***Women*** |  |  |  |  |  |
| Total effect | 0.0326 | 0.0065 | <0.001 | 0.0197 | 0.0454 |
| Direct effect | 0.0326 | 0.0065 | <0.001 | 0.0197 | 0.0454 |
| Indirect effect | 0.0000 | 0.0004 |  | -0.0008 | 0.0008 |
| ***Men*** |  |  |  |  |  |
| Total effect | 0.0214 | 0.0068 | 0.0018 | 0.0081 | 0.0348 |
| Direct effect | 0.0239 | 0.0067 | 0.0004 | 0.0107 | 0.0371 |
| Indirect effect | -0.0025 | 0.0020 |  | -0.0070 | 0.0008 |

b=effect size; SE=standard error; CI=confidence interval

chemerin

BMI

Score_hunger

a= -0.0015

b= -0.0157

c= 0.0326***

c´= 0.0326***

**Online Resource 12a**: Mediation model between chemerin, eating behavior trait “hunger“ and BMI for **women** of the Sorbs cohort.

* P< 0.05, ** P< 0.01, *** P< 0.001; c= total effect; c´= direct effect; a, b= regression coefficient.

chemerin

BMI

Score_hunger

a= -0. 0092

b= 0.2682 **

c= 0.0214**

c´= 0. 0239**

**Online Resource 12b**: Mediation model between chemerin, eating behavior trait “hunger“ and BMI for **men** of the Sorbs cohort.

* P< 0.05, ** P< 0.01, *** P< 0.001; c= total effect; c´= direct effect; a, b= regression coefficient.

**Online Resource 13**: Mediation model between chemerin and “disinhibition” on BMI for women and men of the Sorbs cohort.

|  | **b** | **SE** | **P value** | **Bootstrap 95% CI** | |
| --- | --- | --- | --- | --- | --- |
| ***Women*** |  |  |  |  |  |
| Total effect | 0.0326 | 0.0065 | <0.001 | 0.0197 | 0.0454 |
| Direct effect | 0.0329 | 0.0064 | <0.001 | 0.0203 | 0.0455 |
| Indirect effect | -0.0003 | 0.0013 |  | -0.0030 | 0.0024 |
| ***Men*** |  |  |  |  |  |
| Total effect | 0.0214 | 0.0068 | 0.0018 | 0.0081 | 0.0348 |
| Direct effect | 0.0223 | 0.0066 | 0.0009 | 0.0093 | 0.0353 |
| Indirect effect | -0.0009 | 0.0019 |  | -0.0049 | 0.0029 |

b=effect size; SE=standard error; CI=confidence interval

chemerin

BMI

Score_disinhibition

a= -0.0010

b= 0. 2983***

c= 0.0326***

c´= 0.0329***

**Online Resource 13a**: Mediation model between chemerin, eating behavior trait “disinhibition“ and BMI for **women** of the Sorbs cohort.

* P< 0.05, ** P< 0.01, *** P< 0.001; c= total effect; c´= direct effect; a, b= regression coefficient.

chemerin

BMI

Score_disinhibition

a= -0. 0029

b= 0.3002 ***

c= 0.0214**

c´= 0. 0223***

**Online Resource 13b**: Mediation model between chemerin, eating behavior trait “disinhibition“ and BMI for **men** of the Sorbs cohort.

* P< 0.05, ** P< 0.01, *** P< 0.001; c= total effect; c´= direct effect; a, b= regression coefficient.

**Online Resource 14**: Mediation model between fibroblast-growth factor (FGF)-19 and “hunger” on BMI for women and men of the Sorbs cohort.

|  | **b** | **SE** | **P value** | **Bootstrap 95% CI** | |
| --- | --- | --- | --- | --- | --- |
| ***Women*** |  |  |  |  |  |
| Total effect | -0.0024 | 0.0013 | 0.0639 | -0.0048 | 0.0001 |
| Direct effect | -0.0023 | 0.0013 | 0.0689 | -0.0049 | 0.0002 |
| Indirect effect | 0.0000 | 0.0001 |  | -0.0003 | 0.0002 |
| ***Men*** |  |  |  |  |  |
| Total effect | 0.0010 | 0.0010 | 0.3107 | -0.0010 | 0.0031 |
| Direct effect | 0.0013 | 0.0010 | 0.2150 | -0.0008 | 0.0033 |
| Indirect effect | -0.0003 | 0.0002 |  | -0.0008 | 0.0001 |

b=effect size; SE=standard error; CI=confidence interval

FGF-19

BMI

Score_hunger

a= 0.0007

b= -0.0199

c= -0.0024

c´= -0.0023

**Online Resource 14a:** Mediation model between FGF-19, eating behavior trait “hunger“ and BMI for **women** of the Sorbs cohort.

* P< 0.05, ** P< 0.01, *** P< 0.001; c= total effect; c´= direct effect; a, b= regression coefficient.

FGF-19

BMI

Score_hunger

a= -0.0013

b= 0.1932

c= 0.0010

c´= 0.0013

**Online Resource 14b:** Mediation model between FGF-19, eating behavior trait

“hunger“ and BMI for **men** of the Sorbs cohort.

* P< 0.05, ** P< 0.01, *** P< 0.001; c= total effect; c´= direct effect; a, b= regression coefficient.

**Online Resource 15**: Mediation model between pro-neurotensin (pro-NT) and “hunger” on BMI for women and men of the Sorbs cohort.

|  | **b** | **SE** | **P value** | **Bootstrap 95% CI** | |
| --- | --- | --- | --- | --- | --- |
| ***Women*** |  |  |  |  |  |
| Total effect | -0.0008 | 0.0057 | 0.8878 | -0.0119 | 0.0103 |
| Direct effect | -0.0007 | 0.0057 | 0.8978 | -0.0119 | 0.0104 |
| Indirect effect | -0.0001 | 0.0004 |  | -0.0009 | 0.0007 |
| ***Men*** |  |  |  |  |  |
| Total effect | -0.0050 | 0.0035 | 0.1503 | -0.0118 | 0.0018 |
| Direct effect | -0.0031 | 0.0038 | 0.4095 | -0.0106 | 0.0044 |
| Indirect effect | -0.0019 | 0.0013 |  | -0.0050 | 0.0000 |

b=effect size; SE=standard error; CI=confidence interval

pro-NT

BMI

Score_hunger

a= 0.0016

b= -0.0426

c= - 0.0008

c´= - 0. 0007

**Online Resource 15a:** Mediation model between pro-NT, eating behavior trait “hunger“ and BMI for **women** of the Sorbs cohort.

* P< 0.05, ** P< 0.01, *** P< 0.001; c= total effect; c´= direct effect; a, b= regression coefficient.

pro-NT

BMI

Score_hunger

a= -0.0076 *

b= -0.2457*

c= -0.0050

c´= - 0.0031

**Online Resource 15b:** Mediation model between pro-NT, eating behavior trait “hunger“ and BMI for **men** of the Sorbs cohort.

* P< 0.05, ** P< 0.01, *** P< 0.001; c= total effect; c´= direct effect; a, b= regression coefficient.

**Online Resource 16**: Mediation model between adiponectin and “hunger” on BMI for women and men of the Sorbs cohort.

|  | **b** | **SE** | **P value** | **Bootstrap 95% CI** | |
| --- | --- | --- | --- | --- | --- |
| ***Women*** |  |  |  |  |  |
| Total effect | -0.1501 | 0.0621 | 0.0162 | -0.2722 | -0.0280 |
| Direct effect | -0.1506 | 0.0629 | 0.0172 | -0.2743 | -0.0269 |
| Indirect effect | 0.0005 | 0.0045 |  | -0.0086 | 0.0112 |
| ***Men*** |  |  |  |  |  |
| Total effect | -0.0443 | 0.0436 | 0.3114 | -0.1303 | 0.0418 |
| Direct effect | -0.0357 | 0.0451 | 0.4292 | -0.1246 | 0.0532 |
| Indirect effect | -0.0086 | 0.0090 |  | -0.0292 | 0.0058 |

b=effect size; SE=standard error; CI=confidence interval

adiponectin

BMI

Score_hunger

a= -0.0126

b= -0.0398

c= - 0.1501*

c´= - 0.1506*

**Online Resource 16a:** Mediation model between adiponectin, eating behavior trait “hunger“ and BMI for **women** of the Sorbs cohort.

* P< 0.05, ** P< 0.01, *** P< 0.001; c= total effect; c´= direct effect; a, b= regression coefficient.

adiponectin

BMI

Score_hunger

a= -0.0580

b= -0.1476

c= -0.0443

c´= -0.0357

**Online Resource 16b:** Mediation model between adiponectin, eating behavior trait “hunger“ and BMI for **men** of the Sorbs cohort.

* P< 0.05, ** P< 0.01, *** P< 0.001; c= total effect; c´= direct effect; a, b= regression coefficient.

**Online Resource 17**: Mediation model between fibroblast-growth factor (FGF)-21 and “cognitive restraint” on BMI for women and men of the Sorbs cohort.

|  | **b** | **SE** | **P value** | **Bootstrap 95% CI** | |
| --- | --- | --- | --- | --- | --- |
| ***Women*** |  |  |  |  |  |
| Total effect | 0.0001 | 0.0013 | 0.9560 | -0.0025 | 0.0026 |
| Direct effect | -0.0001 | 0.0013 | 0.9673 | -0.0026 | 0.0025 |
| Indirect effect | 0.0001 | 0.0002 |  | -0.0001 | 0.0008 |
| ***Men*** |  |  |  |  |  |
| Total effect | 0.0021 | 0.0010 | 0.0388 | 0.0001 | 0.0040 |
| Direct effect | 0.0021 | 0.0009 | 0.0248 | 0.0003 | 0.0040 |
| Indirect effect | 0.0000 | 0.0001 |  | -0.0002 | 0.0003 |

b=effect size; SE=standard error; CI=confidence interval

FGF 21

BMI

Score_cognitive restraint

a= 0.0007

b= 0.1810 ***

c= 0.0001

c´= - 0.0001

**Online Resource 17a:** Mediation model between FGF-21, eating behavior trait “cognitive restraint“ and BMI for **women** of the Sorbs cohort.

* P< 0.05, ** P< 0.01, *** P< 0.001; c= total effect; c´= direct effect; a, b= regression coefficient.

FGF 21

BMI

Score_cognitive restraint

a= -0. 0005

b= 0.0889

c= 0. 0021*

c´= 0. 0021*

**Online Resource 18b:** Mediation model between FGF-21, eating behavior trait “cognitive restraint“ and BMI for **men** of the Sorbs cohort.

* P< 0.05, ** P< 0.01, *** P< 0.001; c= total effect; c´= direct effect; a, b= regression coefficient.

**Online Resource 18**: Mediation model between progranulin and “cognitive restraint” on BMI for women and men of the Sorbs cohort.

|  | **b** | **SE** | **P value** | **Bootstrap 95% CI** | |
| --- | --- | --- | --- | --- | --- |
| ***Women*** |  |  |  |  |  |
| Total effect | 0.0201 | 0.0088 | 0.0223 | 0.0029 | 0.0373 |
| Direct effect | 0.0224 | 0.0086 | 0.0098 | 0.0054 | 0.0393 |
| Indirect effect | -0.0023 | 0.0021 |  | -0.0069 | 0.0016 |
| ***Men*** |  |  |  |  |  |
| Total effect | 0.0027 | 0.0108 | 0.8035 | -0.0186 | 0.0240 |
| Direct effect | 0.0042 | 0.0109 | 0.6986 | -0.0173 | 0.0257 |
| Indirect effect | -0.0015 | 0.0016 |  | -0.0052 | 0.0011 |

b=effect size; SE=standard error; CI=confidence interval

progranulin

BMI

Score_cognitive restraint

a= -0.0114

b= 0.2001***

c= 0.0201*

c´= 0.0224**

**Online Resource 18a:** Mediation model between progranulin, eating behavior trait “cognitive restraint“ and BMI for **women** of the Sorbs cohort.

* P< 0.05, ** P< 0.01, *** P< 0.001; c= total effect; c´= direct effect; a, b= regression coefficient.

progranulin

BMI

Score_cognitive restraint

a= -0. 0231*

b= 0.0666

c= 0. 0027

c´= 0.0042

**Online Resource 18b:** Mediation model between progranulin, eating behavior trait “cognitive restraint“ and BMI for **men** of the Sorbs cohort.

* P< 0.05, ** P< 0.01, *** P< 0.001; c= total effect; c´= direct effect; a, b= regression coefficient.

**Online Resource 19**: Mediation model between leptin and “Cognitive restraint” on BMI for women and men of the Sorbs cohort.

|  | **b** | **SE** | **P value** | **Bootstrap 95% CI** | |
| --- | --- | --- | --- | --- | --- |
| ***Women*** |  |  |  |  |  |
| Total effect | 0.2444 | 0.0151 | <0.001 | 0.2147 | 0.2742 |
| Direct effect | 0.2407 | 0.0153 | <0.001 | 0.2106 | 0.2709 |
| Indirect effect | 0.0037 | 0.0031 |  | -0.0017 | 0.0107 |
| ***Men*** |  |  |  |  |  |
| Total effect | 0.1830 | 0.0362 | <0.001 | 0.1092 | 0.2569 |
| Direct effect | 0.1811 | 0.0372 | <0.001 | 0.1050 | 0.2572 |
| Indirect effect | 0.0019 | 0.008 |  | -0.0147 | 0.0232 |

b=effect size; SE=standard error; CI=confidence interval

leptin

BMI

Score_cognitive_

restraint

a= 0.0638**

b= 0.0580

c= 0.2444***

c´= 0.2407***

c´=

**Online Resource 19a:** Mediation model between leptin, eating behavior trait “cognitive restraint“ and BMI for **women** of the Sorbs cohort.

* P< 0.05, ** P< 0.01, *** P< 0.001; c= total effect; c´= direct effect; a, b= regression coefficient.

leptin

BMI

Score_cognitive restraint

a= 0.0311

b= 0.0627

c= 0.1830***

c´= 0.1811***

**Online Resource 19b:** Mediation model between leptin, eating behavior trait “cognitive restraint“ and BMI for **men** of the Sorbs cohort.

* P< 0.05, ** P< 0.01, *** P< 0.001; c= total effect; c´= direct effect; a, b= regression coefficient.

**Online Resource 20**: Mediation model between pro-enkephalin (PENK) and “hunger” on BMI for women and men of the LIFE Adult cohort.

|  | **b** | **SE** | **P value** | **Bootstrap 95% CI** | |
| --- | --- | --- | --- | --- | --- |
| ***Women*** |  |  |  |  |  |
| Total effect | -0.0187 | 0.0099 | 0.0599 | -0.0382 | 0.0008 |
| Direct effect | -0.0161 | 0.0099 | 0.1038 | -0.0355 | 0.0033 |
| Indirect effect | -0.0026 | 0.0016 |  | -0.0058 | 0.0004 |
| ***Men*** |  |  |  |  |  |
| Total effect | -0.0073 | 0.0095 | 0.4419 | -0.0260 | 0.0113 |
| Direct effect | -0.0073 | 0.0101 | 0.4705 | -0.0271 | 0.0125 |
| Indirect effect | 0.0000 | 0.0008 |  | -0.0012 | 0.0020 |

b=effect size; SE=standard error; CI=confidence interval

PENK

BMI

Score_hunger

a= -0.0071

b= 0.3643***

c= -0.0187

c´= -0.0161 ***

**Online Resource 20a:** Mediation model between PENK, eating behavior trait “hunger“ and BMI for **women** of the LIFE Adult cohort.

* P< 0.05, ** P< 0.01, *** P< 0.001; c= total effect; c´= direct effect; a, b= regression coefficient.

PENK

BMI

Score_hunger

a= -0.0001

b= 0.2191***

c= -0.0073

c´= -0.0073

**Online Resource 20b:** Mediation model between PENK, eating behavior trait “hunger“ and BMI for **men** of the LIFE Adult cohort.

* P< 0.05, ** P< 0.01, *** P< 0.001; c= total effect; c´= direct effect; a, b= regression coefficient.

**Online Resource 21**: Mediation model between vaspin and “disinhibition” on BMI for women and men of the LIFE Adult cohort.

|  | **b** | **SE** | **P value** | **Bootstrap 95% CI** | |
| --- | --- | --- | --- | --- | --- |
| ***Women*** |  |  |  |  |  |
| Total effect | -0.4393 | 0.1546 | 0.0046 | -0.7427 | -0.1359 |
| Direct effect | -0.4053 | 0.1504 | 0.0072 | -0.7005 | -0.1100 |
| Indirect effect | -0.0340 | 0.0611 |  | -0.1528 | 0.0860 |
| ***Men*** |  |  |  |  |  |
| Total effect | 0.5326 | 0.2005 | 0.0080 | 0.1390 | 0.9261 |
| Direct effect | 0.5154 | 0.1867 | 0.0059 | 0.1490 | 0.8818 |
| Indirect effect | 0.0172 | 0.0474 |  | -0.0666 | 0.1204 |

b=effect size; SE=standard error; CI=confidence interval

vaspin

BMI

Score_disinhibition

a= -0.0594

b= 0.5734***

c= -0.4393**

c´= -0.4053**

**Online Resource 21a:** Mediation model between vaspin, eating behavior trait “disinhibition“ and BMI for **women** of the LIFE Adult cohort.

* P< 0.05, ** P< 0.01, *** P< 0.001; c= total effect; c´= direct effect; a, b= regression coefficient.

**Online Resource 21b:** Mediation model between vaspin, eating behavior trait “disinhibition“ and BMI for men of the LIFE Adult cohort.

vaspin

BMI

Score_disinhibition

a= 0.0380

b= 0.4516***

c= 0.5326**

c´= 0.5154**

* P< 0.05, ** P< 0.01, *** P< 0.001; c= total effect; c´= direct effect; a, b= regression coefficient.

**Online Resource 22**: Mediation model between vaspin and “cognitive restraint” on BMI for women and men of the LIFE Adult cohort.

|  | **b** | **SE** | **P value** | **Bootstrap 95% CI** | |
| --- | --- | --- | --- | --- | --- |
| ***Women*** |  |  |  |  |  |
| Total effect | -0.4418 | 0.1681 | 0.0087 | -0.7718 | -0.1119 |
| Direct effect | -0.4775 | 0.1689 | 0.0048 | -0.8089 | -0.1460 |
| Indirect effect | 0.0356 | 0.0314 |  | -0.0236 | 0.1011 |
| ***Men*** |  |  |  |  |  |
| Total effect | 0.4783 | 0.2044 | 0.0195 | 0.0772 | 0.8794 |
| Direct effect | 0.4807 | 0.2040 | 0.0187 | 0.0803 | 0.8810 |
| Indirect effect | -0.0024 | 0.0148 |  | -0.0303 | 0.0306 |

b=effect size; SE=standard error; CI=confidence interval

vaspin

BMI

Score_cognitive restraint

a= 0.2328

b= 0.1530***

c= -0.4418**

c´= -0.4775**

**Online Resource 22a:** Mediation model between vaspin, eating behavior trait “cognitive restraint“ and BMI for **women** of the LIFE Adult cohort.

* P< 0.05, ** P< 0.01, *** P< 0.001; c= total effect; c´= direct effect; a, b= regression coefficient.

vaspin

BMI

Score_cognitive restraint

a= -0.0364

b= 0.0650*

c= 0.4783*

c´= 0.4807*

**Online Resource 22b:** Mediation model between vaspin, eating behavior trait “cognitive restraint“ and BMI for **men** of the LIFE Adult cohort.

* P< 0.05, ** P< 0.01, *** P< 0.001; c= total effect; c´= direct effect; a, b= regression coefficient.
